# Quorum sensing in bacteria of rice rhizospheres from Chhattisgarh, India

**DOI:** 10.6026/97320630019199

**Published:** 2023-02-28

**Authors:** Pankaj Kumar, Shivali Sharma, Vibhay Nath Tripathi

**Affiliations:** 1 Department of Botany, Guru Ghasidas University, Bilaspur, India 495009

**Keywords:** bacteria, Quorum sensing, AHL). Rhizospheric

## Abstract

Quorum sensing is a phenomena used by bacteria for regulation of some important characteristics coordinately. Gram-negative and Gram-positive bacteria, both utilize this system of communication, but they use different signal molecules for this, i.e. Gram-negative bacteria preferentially use acyl homoserine lactone (AHL). Rhizospheric soil is rich in bacterial diversity, with the presence of both types of bacteria. It will be useful if we know the quorum sensing based communications among bacteria present in rhizospheric soil. This work was undertaken with the idea to explore the diversity of AHL producing bacteria in soil. For this, bacteria were isolated from rice rhizosphere from field located in Dhamtari district of Chhattisgarh. To study quorum sensing, detection of AHL production was performed by colorimetry method. Out of total 29 bacterial isolates, only three Gram-negative bacteria showed positive result for AHLs production. These 3 isolates were selected for further study. The isolates were characterized by biochemical test. Other characteristics of these bacteria were also tested, like antibiotic resistance profile, indole test, and capability to biofilm formation, phosphate solubilization as well as their growth in different specific culture media. The Results suggest that AHLs producing bacteria may belong to family of *Pseudomonas* sp., which are reported in different researches to be part of the PGPR group of organisms. This work forms the base to study the cell-cell communication in plant rhizospheres.

##  Background:

Quorum sensing (QS) is a cell density dependent communication process used by bacteria during specific group behavior. Specific signal molecules are important for quorum sensing, required in a threshold concentration to start the regulation of various characteristic such as light production, antibiotic production etc. [1]. Quorum sensing signal molecules are small chemicals released by bacteria into extracellular environment during quorum sensing process, which bind to its cognate receptor and finally the signal/receptor complex regulate the target genes. It is first reported in Gram-negative marine bacteria *Vibrio fischeri*, and it has luxI/luxR quorum sensing system (i.e. luxI encodes signal synthase and luxR encodes signal receptor) [2]. On the basis of chemical structure and action mechanism of signal molecules, they belong to one of three classes: first one is acyl homoserine lactone, commonly used by the Gram-negative bacteria. Second class of signal molecule: oligopeptides or its derivatives are majorly used by Gram-positive bacteria, while the third signal molecules known as autoinducer-2 (AI-2) used by both Gram-negative and Gram-positive bacteria [3]. Recently other class of signal molecules are also reported, like -Autoinducer-3 (AI-3) in enterohemorrhagic E. coli. (EHEC), HHQ (2-heptyl-4(1H)-quinolone) and PQS (2-heptyl-3-hydroxyl-4-(1H)-quinolone), DSF (diffusible signal factor), IQS (integrator of quorum sensing) and CDP (cyclic dipeptide) etc. in *Pseudomonas aeruginosa* [4, 5]. AHLs are made up of two components namely, fatty acyl chain and lactone ring. Diversification of AHLs is based on the acyl side chain, which varies in their degree of saturation, oxidation and substitution at third carbon of chain and also in their carbon length that ranges from C4 to C18 [6]. Rhizospheric soil is very rich in its micronutrient contents and that's why it harbors a large number of bacteria. Both Gram-negative and Gram-positive rhizobacteria use density dependent mechanism for interesting communication and regulation of multiple aspects of their life, i.e. antibiotic production, motility, sporulation, symbiotic association, virulence, competence, conjugation and biofilm formation etc. [7]. Lots of work has been done in study of diversity of bacteria in rhizospheric soil and their role in plant improvement, but only few literatures are available for quorum sensing capabilities of these bacteria. Balasundararajan and Dananjeyan (2019) reported that rice rhizospheric QS positive bacteria which can form biofilm [8]. It is also reported that C4- AHLs producing bacteria are best for biofilm formation. AHLs extracted from bacteria are also reported to affect the seedling growth and development, especially on root hair formation [9]. It is reported that α-, β-, γ- proteobacteria can produce AHL, and among them, some common genera are *Rhizobium*, *SinoRhizobium*, *Pseudomonas*, Variovorax, Sphingomonas, Massilia, Aeromonas, Polymorphu, Agrobacterium, Ensifer and Pectobacterium reported from different rhizosphere habitats [10, 11]. The present study was designed to explore the bacterial diversity in rice rhizosphere, in reference to quorum sensing. To the best of our knowledge, it is the first such report from Chhattisgarh. These findings will help in understanding the interactions among diversified bacteria in reference to quorum sensing. This information can also be used to further explore the possibility of using these AHL-positive bacteria for rice improvement in Chhattisgarh.

## Material and Methods:

## Sample collection and study area:

Rice rhizospheric soil samples were collected from rice field, which is located at Dhamtari district of Chhattisgarh, India. At the time of collection, rice crop was in grain settled stage and age of rice plant was 100 days. Three samples were collected randomly from same field. Rice plant was uprooted to collect the rhizospheric soil and collected into sterile poly bags. The samples were stored at 4°C till further use.

## Isolation and purification of bacteria:

Rice rhizospheric bacteria were isolated by standard serial dilution protocol. Briefly, 1 gm of soil dissolved into total of 10 ml of 0.9% sterile saline, and was properly mixed with the help of vortex. 1 ml of suspended soil was transferred into next test tube filled with 9 ml of saline water and make 10-1 dilution, and this step was continued till 10-5 dilution. 200 µl of 10-3 to 10-5 diluted sample were spread on large (150 mm) nutrient agar plate, in three sets. These plates were incubated at room temperature for 24 hours and appearance of different colonies was recorded, on the basis of morphology, texture etc. as well as CFUs (colony forming unit) was also counted for calculation of total number of bacteria in soil sample. Different isolates were selected for further study on the basis of their different physical characteristics. Pure cultures were prepared and glycerol stocks were kept for long term storage.

## Gram staining test:

All isolated rhizospheric bacteria were characterized by Gram staining test and microscopic studies. The standard Gram staining protocol was followed, then, prepared slides were observed under light microscope (OPTIKA microscopes Italy, B-383PHI). Gram-negative bacteria look pink/red, while Gram-positive bacteria appear blue/purple.

## Detection of AHLs:

All purified rice rhizospheric bacteria were tested for the presence of AHLs.

## Extraction of AHLs:

Liquid-liquid extraction processes were done with chloroform. Briefly, isolated bacterial cultures were inoculated into 10 ml LB broth medium with shaking at room temperature for 48 hours. After incubation, cultures were centrifuged for 10 min at 12,000 rpm and the supernatants were collected into fresh test tubes for liquid-liquid extraction. Collected cell free supernatants (CFS) were extracted twice with equal volume of chloroform (HiMedia), as described earlier Yang et al. 2006 [12]. Then the organic phases were taken into a beaker and chloroform was evaporated, into a water bath (40-45deg;C). Evaporated extracts were re-dissolved in 1 ml of 20% acetonitrile, and stored at -20°C until use.

## Colorimetry and spectrometry observation:

Sample dissolved in 20% acetonitrile was used to confirm the presence of AHL as described by Yang et al. 2006 [12], which detects the lactone ring of AHL molecules. Briefly, 1.25 ml of solution-1 (mixture of 2M hydroxylamine hydrochloride and 3.5M sodium hydroxide, 1:1 ratio) were mixed into 1 ml of dissolved extract. After one minute, 1 ml of solution-2 (mixture of 95% ethanol and 10% ferric chloride into 4M HCL, 1:1 ratio) is mixed. Presence of AHL is shown by appearance of dark brown color in solution, which was further quantified by spectrophotometric method (absorbance reading at 520 nm in SYSTONICS Double Beam Spectrophotometer). As per the spectrometric observations (i.e. absorbance value of more than 1), the bacterial isolates were selected for further studies.

## Physiological and biochemical test used for the identification and characterization of isolates:

## Growth in Different media:

## MacConkey agar:

This media is used as a selective and differential culture media for isolation of Gram-negative and enteric bacteria. Pure cultures of isolates were streaked onto prepared MacConkey agar (Himedia) plate and incubated for 48hours at 37 °C. After incubation, isolates grown on MacConkey agar plates considered as Gram-negative bacteria.

## Pikovskaya's agar:

For the test of phosphate solubilizing activity of isolates, we used Pikovskaya's medium. Pure cultures of selected isolates were spot-inoculated on the prepared Pikovskaya's agar plates and incubated at 30 °C for 5 days. The appearances of clear zone around bacterial growth were considered as positive result for phosphate solubilization activity [13].

## King's B media:

Selected isolates were grown into King's B medium plates, which is commonly used for isolation of *Pseudomonas* and for studying fluorescence production. Pure culture of selected isolates were streaked on prepared King's B media plate and incubated for one week at 37 °C then fluorescence production was observed under UV light (360 nm) [14].

## Congo red agar:

Congo red agar media is used to study the capability to biofilm formation, as described by Freeman et al. (1989). Fresh culture of isolates were streaked into prepared Congo red plates and incubated for 24-48 hours at 37°C. Black colonies with a dry crystalline consistency considered as positive result [15].

## Disk diffusion assay:

Standardized protocol of Kirby-Bauer disk diffusion susceptibility test was applied to selected isolates. For this purpose, we used total 8 readymade antibiotic disks with known concentration namely Azithromycin (30 mcg), Vancomycin (30 mcg), Rifampicin (5 mcg), Gentamicin (10 mcg), Streptomycin (25 mcg), Levofloxacin (5 mcg), Ampicilin (10 mcg) and Penicillin (10 units). Briefly, 200 µl of overnight grown culture was spread on prepared nutrient agar plates and incubated for approx. 1 hour. After incubation, antibiotic disks were placed on plates and further incubated for 24 hours at 37°C. After incubation clear zone around the disk show sensitivity. The zone diameter was measured. No such clear zone indicates the resistance against antibiotics [16].

## Biochemical and other test:

Biochemical tests of the selected isolates were performed as per the protocols described by Bergey (1994) [17]. Important tests, which were performed, are Urease test, Catalase test, Oxidase test, Gelatin hydrolysis test, Glucose and lactose fermentation test etc. Indole test was also performed according to the protocol given by Maria et al. (2008) for the detection of Indole production [18].

## Results:

## Morphological and microscopic studies:

**Figure 1:**
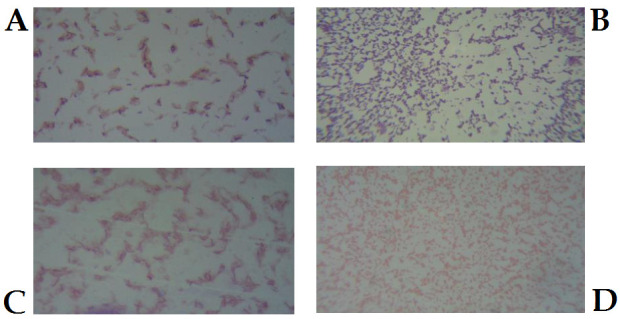
Representative pictures of microscopic observation. Gram-Positive Bacteria- A (SA7) and B (SA11), Gram-negative Bacteria- C (SA3) and D (SA6)

On the basis of morphological studies we isolated total 29 bacteria from the rice rhizospheric soil. As per the microscopic studies among 29 isolates, 7 bacteria were characterized as Gram-negative and 22 bacteria were characterized as Gram-positive. Bacterial shape varied from cocci to bacilli, while their size was also variable. Details of the microscopic studies with size and shape of the isolates are showed in Table 1. Representative pictures of Gram-negative and Gram-positive bacteria are also shown in Figure 1. The microbial density in rice rhizospheric soil was calculated to be approximately 2 x 106cfu/gm soil.

## AHLs detection:

**Figure 2:**
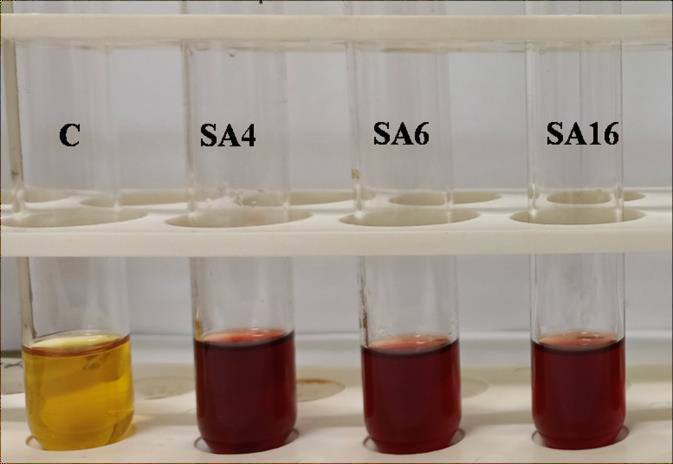
Colorimetry results. Positive results of SA4, SA6, SA16 (dark brown color), C-Control

Each isolates were screened for the presence of AHLs by Colorimetry and spectrometry method. Only three bacteria, namely SA4, SA6 and SA16, showed dark brown color meaning the presence of AHL (Figure 2). High absorbance was also indicative of AHLs positive bacteria (Table 1). Interestingly, all three AHLs positive bacteria were characterized as Gram-negative bacteria by microscopic observations. These AHL positive bacteria were selected for further studies.

## Growth in different media:

**Figure 3:**
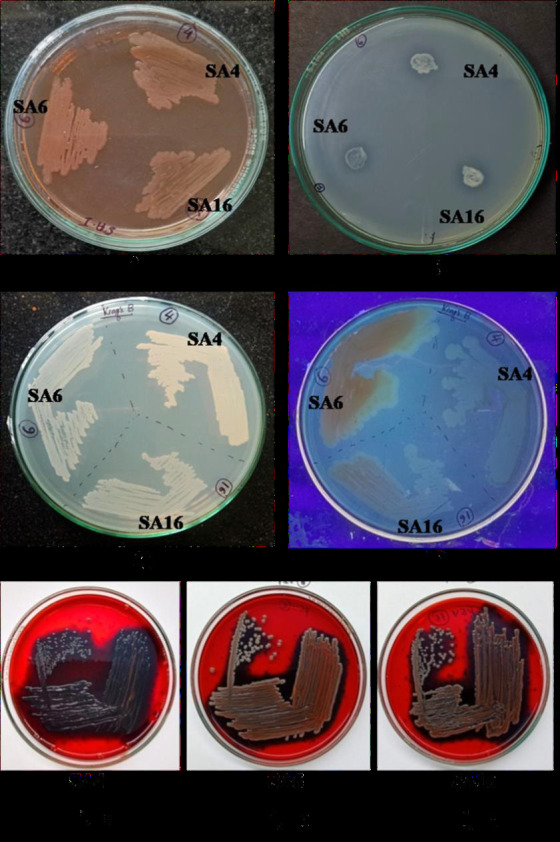
Selected isolates SA4, SA6 and SA16 grown in different media. MacConkey agar (A), Pikovskaya's agar (B), King's B medium (C), King's B medium under UV light (D) and Congo red agar (E. a-E. c).

Selected isolates SA4, SA6 and SA16 were grown in specific media to study their growth behaviour. Strains showed good growth on all the plates i.e. MacConkey agar, Pikovskaya's agar, Congo red agar and King's B agar plates. When these cultures were checked for fluorescence under UV light, all of them were negative (Figure 3).

## Antibiotic disk diffusion assay:

**Figure 4:**
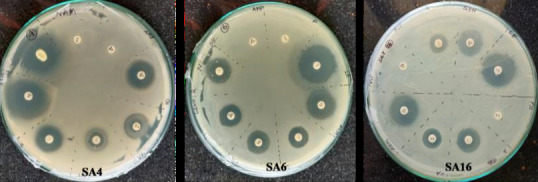
Antibiotic disk diffusion assay. SA4 (A), SA6 (B) and SA16 and (C) details of the antibiotic disks are given in text.

Antibiotic resistance profiling of the strains were also done by disk diffusion assay. Results of the disk diffusion assay with different concentrations of antibiotics used against sample SA4, SA6 and SA16, showed that these organisms are resistance against Ampicillin and Penicilin, whereas all of them are sensitive towards Levofloxacin, Azithromycin, Streptomycin, Vancomycin, Rifampicin and Gentamicin. Among 6 antibiotics for which the strains are sensitive, Levoflxocin had highest impact as shown by largest zone of inhibition and Vancomycin had lowest impact as was evident from lowest size of zone inhibition (Figure 4).

## Biochemical test:

**Figure 5:**
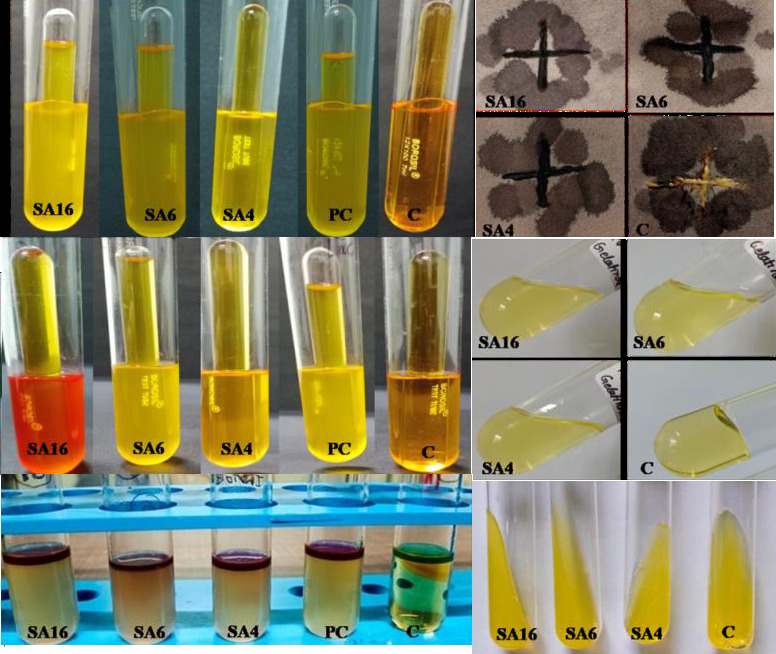
Biochemical and other test. Glucose fermentation test (A), Oxidase test (streaked as plus sign) (B), Lactose fermentation test (C), Gelatin hydrolysis test (D), Indole test (E) and Urease test (F). C- Control, PC- Positive control.

Selected isolates SA4, SA6 and SA16 were further characterized by various biochemical tests for help in their identification. Results of these biochemical tests are presented in Table 2. All three selected isolates showed positive result for the Indole, Catalase, Gelatin hydrolysis and Oxidase test while negative result for Urease test. Based on the Carbohydrate fermentation reaction bacteria are classified as: fermenter with acid production only, fermenter with both acid and gas production and non-fermenter bacteria. Glucose fermentation test indicated that SA4 is a fermenter with acid production only, while SA6 and SA16 are classified as fermenter with both acid and gas production. Result of lactose fermentation test showed SA4 as a fermenter with acid production only, SA6 as a fermenter with acid and gas production, while SA16 as non-fermenter bacteria. SA16 showed bubble formation in solution, which may be the result of gas production by other metabolic pathway. Additionally, it is observed that SA16 also showed red color, which may be a result of some kind of pigment production (Figure 5 and Table 2) [19].

## Discussion:

In present study, total 29 bacteria were isolated from rice rhizospheric soil. Among these 29 isolates 3 isolates were identified as AHL producing and characterized as Gram-negative bacteria. To the best of our knowledge, it is a first report on occurrence of AHL producing bacteria isolated from rice field of Chhattisgarh. Diversity of AHL producer bacteria from mangrove associated plant were studied by Vishwanath et al. 2015, and found that 7% of all isolated mangrove rhizobacteria showed positive induction of AHL signals [20]. Our results also showed that approximately 10% of total isolates are AHL producers. In literature, there are some reports on presence of AHL producing bacteria in rice rhizosphere. Steindler et al. 2008 reported AHL-positive *Pseudomonas* from rice rhizospheric soil which is involved in root colonizing [21]. As rhizospheric bacteria are sometime location and plant specific, our result has high significance with reference to the fact that Chhattisgarh is known for rice production. The AHL-producing isolates have shown antibiotic resistance against ampicillin and penicillin, as well as they are positive for biofilm production, indole formation and phosphate solubilization. We speculate that regulation of these characteristics may be connected with AHL signal production based on evidences available in literature. Wang et al. 2012 found that AHL induces biofilm formation in *Pseudomonas* sp. HF-1 [22]. Balasundararajan and Dananjeyan, 2019 studied AHL mediated biofilm formation by bacteria from rice rhizoplane and found that AHL positive bacteria profusely colonized the rice root upon inoculation and formed biofilm on the surface of the root under gnotobiotic conditions. They suggested that biofilm ensure competitive colonization on the rhizoplane and thereby improve plant growth and health [8]. Begum et al. , 2019 also reported rice rhizospheric AHL producing bacteria which can form biofilm [11]. However, the relationship was not studied. Our selected isolates showed resistance against only two antibiotics namely ampicillin and penicillin, when tested against 8 different antibiotics. This may be due to horizontally acquired gene or some changes due to quorum sensing. It may be possible that regulation of antibiotics resistance is linked to quorum sensing. Zhao et al. , mentioned in their paper that QS is sometimes linked to antibiotics resistance activity via efflux pump, biofilm and secretion system [23]. Tamad et al. , 2020 reported that phosphate solubilizing bacteria secreted AHLs and they noticed that butanoyl-AHL (C- 4AHL) was mainly found among all AHLs [24]. Ghosh et al. reported that Burkholderia species uses quorum sensing system to indirectly regulate phosphate solubilization by inducing biofilm formation [25]. Jung et al. studied the relationship between quorum sensing and indole-3-acetic acid (IAA) production in Serratia fonticola GS2 [26]. Biochemical tests were performed for the identification of selected isolates SA4, SA6 and SA16 and results

were indicative that all of these may belong to genus *Pseudomonas* as per Bergey's manual [17]. Elasri et al. also reported that quorum sensing positive *Pseudomonas* found in rice rhizospheric soil and the number of AHL producing *Pseudomonas* species found more in rhizospheric soil as compared to non-rhizosphere soil [27]. Bacterial identification needs to be confirmed by 16S rRNA sequencing. The knowledge gained from this study will be useful for understanding of quorum sensing of bacteria in rhizospheric environment. This study will further open ways for exploration of AHL-regulated different physiological behaviors. These findings may lead to increased rice production, by maneuvering the AHL production in rhizosphere.

## Conclusion:

This study was focused on identification of AHL-producing bacteria from rice rhizospheric soil. Total 3 isolates were found to be AHL positive. They were shown to have some biologically important characteristics like antibiotic resistance, biofilm formation, phosphate solubilizing activity and indole formation etc. The study of quorum sensing is very important to understand the biological interactions among bacterial species present in rhizosphere and to see their effect to the environment.

## Figures and Tables

**Table 1:** Microscopic and spectrometric observation

**S. No.**	**Sample no.**	**Gram staining**	**Shape & size of bacteria**	**Absorbance at 520 nm**
1	SA1	Positive	Streptococcus, small	0.4236
2	SA2	Positive	Bacilli, large	0.423
3	SA3	Negative	Bacilli, small	0.299
4	SA4	Negative	Cocci, small	2.96
5	SA5	Positive	Cocci, small	0.4986
6	SA6	Negative	Bacilli, small	2.96
7	SA7	Positive	Bacilli, large	0.457
8	SA8	Positive	Streptobacillus, large	0.1823
9	SA9	Positive	Streptobacillus, large	0.1213
10	SA10	Positive	Streptobacillus, large	0.4233
11	SA11	Positive	Bacilli, small	0.5613
12	SA12	Positive	Bacilli, large	0.345
13	SA13	Positive	Bacilli, large	0.32
14	SA14	Positive	Streptobacillus, large	0.4023
15	SA15	Negative	Bacilli, large	0.3173
16	SA16	Negative	Bacilli, small	2.96
17	SA17	Positive	Bacilli, large	0.289
18	SA18	Positive	Bacilli, large	0.885
19	SA19	Positive	Streptobacillus, large	0.73
20	SA20	Negative	Bacilli, small	0.69
21	SA21	Positive	Cocci, small	0.163
22	SA22	Positive	Bacilli, large	0.102
23	SA23	Positive	Bacilli, large	0.23
24	SA24	Positive	Bacilli, small	0.476
25	SA25	Positive	Cocci, small	0.124
26	SA26	Negative	Bacilli, large	0.223
27	SA27	Positive	Bacilli, small	0.67
28	SA28	Positive	Streptobacillus, large	0.451
29	SA29	Positive	Bacilli, small	0.364

**Table 2:** Summary of Results of biochemical test

**S. N.**	**Sample no**	**Indole test**	**Catalase test**	**Oxidase test**	**Urease test**	**Glucose fermentation**		L**actose fermentation**	
						Fermentation	Bubble formation	Fermentation	Bubble formation
1	SA4	Positive	Positive	Positive	Negative	Positive	Negative	Positive	Negative
2	SA6	Positive	Positive	Positive	Negative	Positive	Positive	Positive	Positive
3	SA16	Positive	Positive	Positive	Negative	Positive	Positive	Negative	Positive
